# Osteoclasts secrete leukemia inhibitory factor to promote abnormal bone remodeling of subchondral bone in osteoarthritis

**DOI:** 10.1186/s12891-021-04886-2

**Published:** 2022-01-25

**Authors:** Xin Zhao, Long Ma, Haohui Guo, Jian Wang, Shuai Zhang, Xiaochun Yang, Lvlin Yang, Qunhua Jin

**Affiliations:** 1 Department of Orthopedics, General Hospital of Ningxia Medical University, 804 Shengli Street, Yinchuan, Ningxia China; 2 School of Clinical Medicine, Ningxia Medical University, Yinchuan, Ningxia China

**Keywords:** Osteoarthritis, Osteoclasts, Leukemia inhibitory factor, Alendronate, Sclerostin, Abnormal bone remodeling

## Abstract

**Background:**

Osteoarthritis (OA) is a common chronic degenerative joint disease. At present, there is no effective treatment to check the progression of osteoarthritis. Osteochondral units are considered to be one of the most important structures affecting the occurrence and development of osteoarthritis. Osteoclasts mediate an increase in abnormal bone remodeling in subchondral bone in the early stage of osteoarthritis. Here, alendronate (ALN) that inhibit osteoclasts was used to study the regulatory effect of osteoclast-derived leukemia inhibitory factor (LIF) on early abnormal bone remodeling.

**Methods:**

This study involved 10-week-old wild-type female C57BL/6 mice and female *SOST* knockout (KO) mice that were divided into the sham, vehicle, ALN, and *SOST* KO groups.

**Results:**

The expression of LIF was found to decrease by inhibiting osteoclasts, and the histological OA score suggested that the degeneration of articular cartilage was attenuated. Additionally, micro-CT showed that osteoclasts inhibited in the early stage of OA could maintain the microstructure of the subchondral bone. The parameters of bone volume fraction (BV/TV), subchondral bone plate thickness (SBP.Th), and trabecular separation (Tb.Sp) of the treated group were better than those of the vehicle group.

**Conclusions:**

These results suggested that downregulating the expression of sclerostin in osteocytes by secreting LIF from osteoclasts, activate the Wnt/β-catenin signaling pathway, and promote abnormal bone remodeling in OA. Therefore, clastokine LIF might be a potential molecular target to promote abnormal bone remodeling in early OA.

**Supplementary Information:**

The online version contains supplementary material available at 10.1186/s12891-021-04886-2.

## Introduction

Osteoarthritis is a degenerative disease affecting the entire joint in individuals undergoing aging. Assessment of health-related quality of life (HRQOL) in the Chinese population shows that the severity of knee osteoarthritis affects the physical and mental health of patients [1]. The latest report from the United States bone and joint initiative show that the prevalence rate of OA in the past 3 months was 10.4%, which is one of the most common diseases incurring the all-cause medical cost as high as 37.3 billion US dollars [2]. Joint injuries and deformities caused by this disease seriously affect the quality of life of the patients and bring a huge burden to families, government health expenditure, and the social economy. However, at present, the pathogenesis of osteoarthritis is still unclear, and there are no effective treatment measures. Therefore, it is necessary to clarify the pathogenesis of the disease to identify the prevention and treatment methods.

To date, the origin of osteoarthritis whether from the cartilage or subchondral bone is controversial. Traditionally, osteoarthritis begins with the wear and tear of articular cartilage, but recent evidence suggests that subchondral bone remodeling is essential for initiating and leading to the disease progression [3]. The subchondral bone with mechanical support and the bone–cartilage interface with important mechanical properties are mildly damaged due to factors such as abnormal stress. This results in bone remodeling changes and then affects the integrity of the overlying cartilage [4]. The pathological changes in the subchondral bone are more obvious than those of cartilage in the early stage of OA [5].

Bone remodeling is regulated by the interaction between osteoblasts and osteoclasts at the site of injury and signal transduction releasing soluble mediators [6]. The imbalance of coupling bone remodeling is mediated by osteoclasts and osteoblasts. In addition, the migration of T and B cells to the injured site may be involved in osteoclastogenesis and bone catabolism [7]. In the early stage of OA, the activity of osteoclasts and osteoblasts increases by 190 and 96%, respectively [3], which increase bone remodeling and bone loss. With the progress of the disease, the proliferation of osteoblasts and bone remodeling slows down, and OA progression aggravates [5].

LIF is a multifunctional secretory cytokine that participates in bone metabolism [8]. Grimandet al. reported that LIF is not expressed in osteoclasts under physiological conditions [9]. However, Koide et al. reported that osteoclasts mainly secrete LIF, which can inhibit sclerostin and bone turnover [10]. Sclerostin is a negative regulator of Wnt/β-catenin signaling, which is encoded by the *SOST* gene secreted by the osteocytes. The decrease in sclerostin can activate the Wnt signaling pathway and promote bone formation [11]. Genome-wide association studies (GWAS) indicate that the Wnt signaling pathway plays a key role in the progression of osteoarthritis [12]. Therefore, the activation of osteoclasts in the early stage of OA may be the initial factor in the pathogenesis of OA and abnormal bone remodeling of the subchondral bone.

Osteoclast differentiation is induced by macrophage colony-stimulating factor (M-CSF) and receptor activator for nuclear factor-κB ligand (RANKL) [13]. In addition, the osteocytes in bone matrix increase osteoclast production in response to the apoptotic signal from microfractures [14]. Bisphosphonates are commonly used drugs for treating osteoporosis and fracture prevention. They effectively inhibit bone turnover and bone resorption. Mohammad et al. reported that alendronate attenuates the progression of OA in mice [15]. Therefore, this study aimed to investigate the mechanism of attenuating OA progression in an anterior cruciate ligament transection (ACLT) mice model by intraperitoneal injection of alendronate to inhibit osteoclasts. This would provide evidence to support the effect of osteoclast-secreting LIF on the abnormal bone remodeling of the subchondral bone, so as to clarify the pathogenesis of OA and provide the basis for clinical treatment.

## Materials and methods

### Mice used in the study

10-week-old wild-type female C57BL/6 mice were purchased from the Vital River (Beijing, China) and the female *SOST* gene knockout mice were provided by the research group. These animals were raised in SPF animal center, 12/12 h day and night, relative temperature 24–26 °C, relative humidity 50–60%. They were then divided into the sham, vehicle, ALN, and *SOST* KO groups (*n* = 6 in each group). Before the operation, 1% pentobarbital sodium (1.0.ml/ 100 g) was used for intraperitoneal injection anesthesia. The anterior cruciate ligament (ACL) of the right knee was cut through the medial parapatellar approach to establish the OA model. For the sham and *SOST* KO groups, only the ACL of the right knee joint was exposed without cutting off after anesthesia, and the joint capsule and skin were sutured separately. After the operation, the mice were placed in a constant-temperature electric blanket for monitoring until they recovered naturally. The general health status, pain, and infection of mice were monitored daily after the operation. On the second day after ACLT, alendronate sodium (product No. A4978; Sigma-Aldrich; Merck) was injected intraperitoneally twice a week (1000 μg/kg/dose) or vehicle (phosphate-buffered saline) of the same volume. The wild-type mice in the sham, vehicle, and ALN groups were euthanized at 0, 4, and 8 weeks after operation. The mice in *SOST* KO group was euthanized at 4 weeks. All the animal experimental schemes were examined and approved by the medical research ethics committee of the General Hospital of Ningxia Medical University (Yinchuan, China) (protocol No. 2018–056).

### Histochemical study

Following euthanasia, the right knee joint was collected and fixed with 4% paraformaldehyde for 24 h. Then, the specimens were decalcified in EDTA decalcification solution for 4 weeks and then embedded in paraffin. The wax block was fixed on a tissue slicer. The medial compartment was sectioned continuously with a thickness of 4 μm and was used for safranin-O fast green staining,and a histological score was obtained. Dewaxing with two changes of xylene for 10 min. Treating with three changes of 100% absolute ethanol for 3 min, 95% ethanol for 3 min, 70% ethanol for 2 min. Harris Hematoxylin was developed the treating slides for 3 min, then the slides were washed in tap water for 10 min. Sections were stained with 0.2% Fast Green (product No. F7258; Sigma-Aldrich; Merck) for 3 min, and then washed by 1% acetic acid for 5 s, 0.1% Safranin O (product No. S8884; Sigma-Aldrich; Merck) for 3 min. Sections were hydrated in 95% ethanol for 5 s, 100% ethanol for 15 s, fixation by 2 changes in xylene. The Osteoarthritis Research Society International (OARSI) score was used to assess the histopathological grading of cartilage.

### The tartrate-resistant acid phosphatase staining

The tartrate-resistant acid phosphatase (TRAP) staining was performed according to the standard protocol. After the sections were treated with 70% ethanol, the sections were covered with TRAP stain for 30 min. TRAP buffer is produced by acetate anhydrous (product No. S818278; Macklin) 2.05 g, tartrate tetrahydrate (product No. P816440; Macklin) 5.65 g and RO water 500 ml(pH 5.0). TRAP stain is produced by TRAP buffer 50 ml, Naphthol AS-MX Phosphate (product No. N4875; Sigma-Aldrich; Merck) 5 mg, N,N-Dimethylformamide (product No. D4551; Sigma-Aldrich; Merck) 0.5 ml and Fast Red Violet LB Salt (product No. F3381; Sigma-Aldrich; Merck) 20 mg. Harris Hematoxylin was Developped the treating sections for 3 min, then the sections were washed in tap water for 10 min. The slides were dried overnight at 37 °C.

### Immunohistochemical, immunofluorescence, and histomorphometric studies

A standard protocol was used for immunohistochemical staining. Sections were incubated with primary antibody, matrix metalloproteinase 13 (MMP-13) (ab39012, Abcam, 1:400), type X collagen (Col X, ab58632, Abcam, 1:100), LIF (ab138002, Abcam, 1:200), sclerostin (ab63097, Abcam, 1:50), β-catenin (ab16051, Abcam, 1:200), and osteocalcin (ab93876, Abcam, 1:200), overnight at 4 °C. Subsequently, the polymer detection system (ZSGB BIO) was used to detect the immune activity, and then hematoxylin (ZSGB BIO) was used for counterstaining. For immunofluorescence staining, sections were incubated with Alexa Fluor® 488 goat anti-rabbit secondary antibody (ab150077, Abcam, 1:500) for 1 h at 37 °C in the dark. The morphological measurement of the subchondral bone tissue was performed using a Leica microscope (MD3000), the cartilage was evaluated by Zeiss fluorescence microscope (AXIO OBSERVERA1), and the quantitative analysis was performed blindly. For MMP-13 and Col X, the positive staining number and the total number of chondrocytes in the entire articular cartilage were calculated. For TRAP-positive osteoclasts, LIF, sclerostin, β-catenin, and osteocalcin in subchondral bone, three visual fields of each sample were taken for positive cell count and bone surface length measurement for analysis.

### Micro-computed tomography (Micro-CT) analysis

Excess muscle tissues were removed from the entire right knee joint of the mice and fixed in 10% formalin overnight. The specimens were scanned using a microcomputer (Inveon PET.SPECT.CT, Siemens). The images were scanned (Inveon Acquisition Workplace), reconstructed (COBRA_Exxim, Siemens), and further analyzed using the calculation software (Ineon Research Workplace). The X-ray current and voltage were set to 500 μA and 80 kV, respectively, and the resolution was 8.5 μm/μm/pixel. The sagittal view of the entire medial compartment of the tibial subchondral bone was used for the 3D histomorphometry analysis, and the parameters of bone volume/total tissue volume (BV/TV), subchondral bone plate thickness (SBP.Th), and trabecular separation (Tb.Sp) were analyzed.

### Statistics

The data are presented as mean ± SD. Independent sample t test was used to determine the difference between the two groups, and one-way ANOVA was used to determine the differences between different groups. *P* < 0.05 means the difference was statistically significant. All data were analyzed with IBM SPSS 19.0.

## Results

### Articular cartilage preservation by inhibiting osteoclasts

**Fig. 1:**
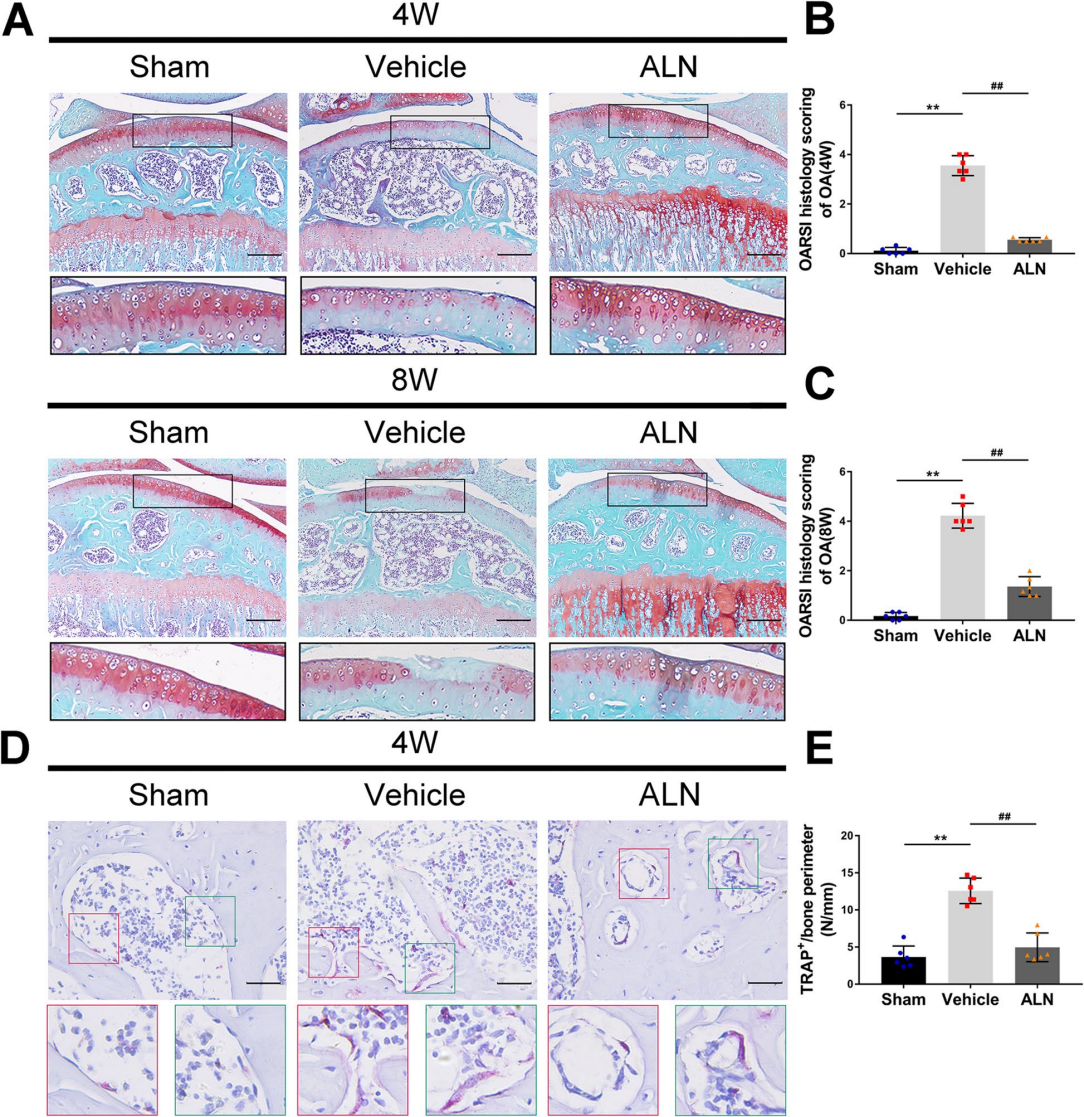
(**A**) Safranin-O fast green staining shows progressive proteoglycan loss and the cartilage damage at 4 and 8 weeks postoperatively. Scale bar, 200 μm. (**B**, **C**) Histologic OA score of the articular cartilage at 4 and 8 weeks. (**D**, **E**) Immunostaining and quantitative analysis for TRAP in sagittal sections of the subchondral bone medial compartment at 4 weeks after ACLT surgery. Scale bar, 50 μm. Vehicle = ACLT surgery treated with vehicle. ALN = ACLT surgery treated with alendronate. Numerical data are presented as means ± SD; ^**^p < 0.01 compared to the sham group, ^##^*p* < 0.01 compared to the vehicle group

**Fig. 2:**
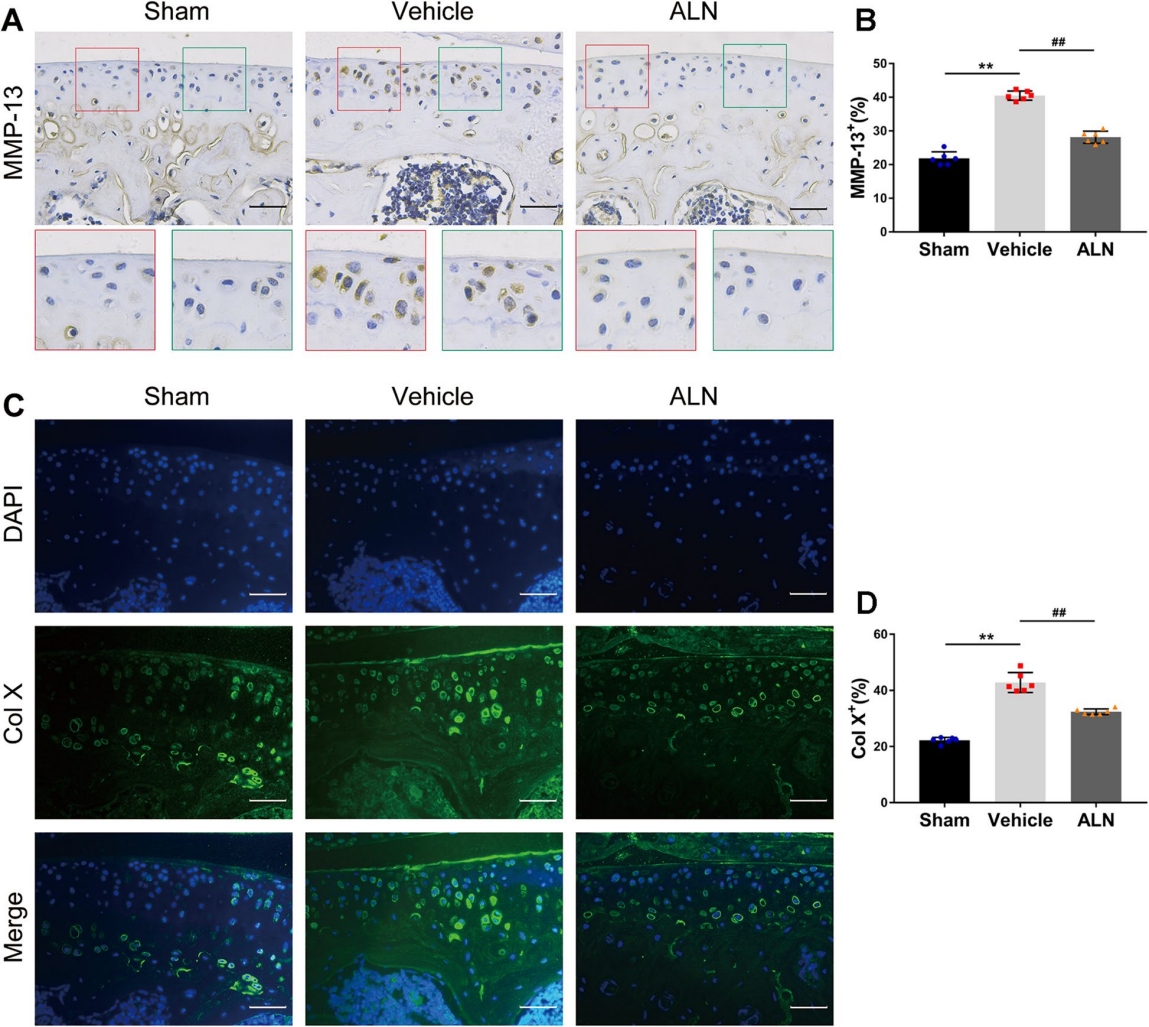
(**A**, **B**) Inhibition of osteoclasts can reduce the ratio of matrix metalloproteinase-13 (MMP-13) positive cells in articular cartilage at 4 weeks after ACLT surgery. Immunostaining and quantitative analysis for MMP-13. (**C**, **D**) Immunofluorescence staining and quantitative analysis for collagen X. Scale bar, 50 μm. Means ± SD; ^**^p < 0.01 compared to the the sham group, ^##^*p* < 0.01 compared to the the vehicle group

First, the Safranin-O and Fast Green staining showed that the loss of proteoglycan in the corresponding ALN group was significantly reduced compared to that in the vehicle group 4 and 8 weeks after the operation (Fig. 1 A). The severity of cartilage degeneration was assessed using the OARSI histological score. After inhibition of osteoclast activity, the score of the ALN group was significantly lower than that of the vehicle group at 4 and 8 weeks after the operation (Fig. 1 B, C). The results of TRAP staining at 4 weeks confirmed that ALN could effectively inhibit osteoclasts (Fig. 1 D, E). Immunohistochemistry results showed that compared with the vehicle group, the number of MMP-13 positive cells in the ALN group decreased the injury of chondrocytes (Fig. 2 A, B). Col X data also showed similar results (Fig. 2 C, D). These results suggest that the inhibition of osteoclasts attenuated the degeneration of the articular cartilage.

### Maintenance of subchondral bone microstructure by inhibiting osteoclasts

**Fig. 3:**
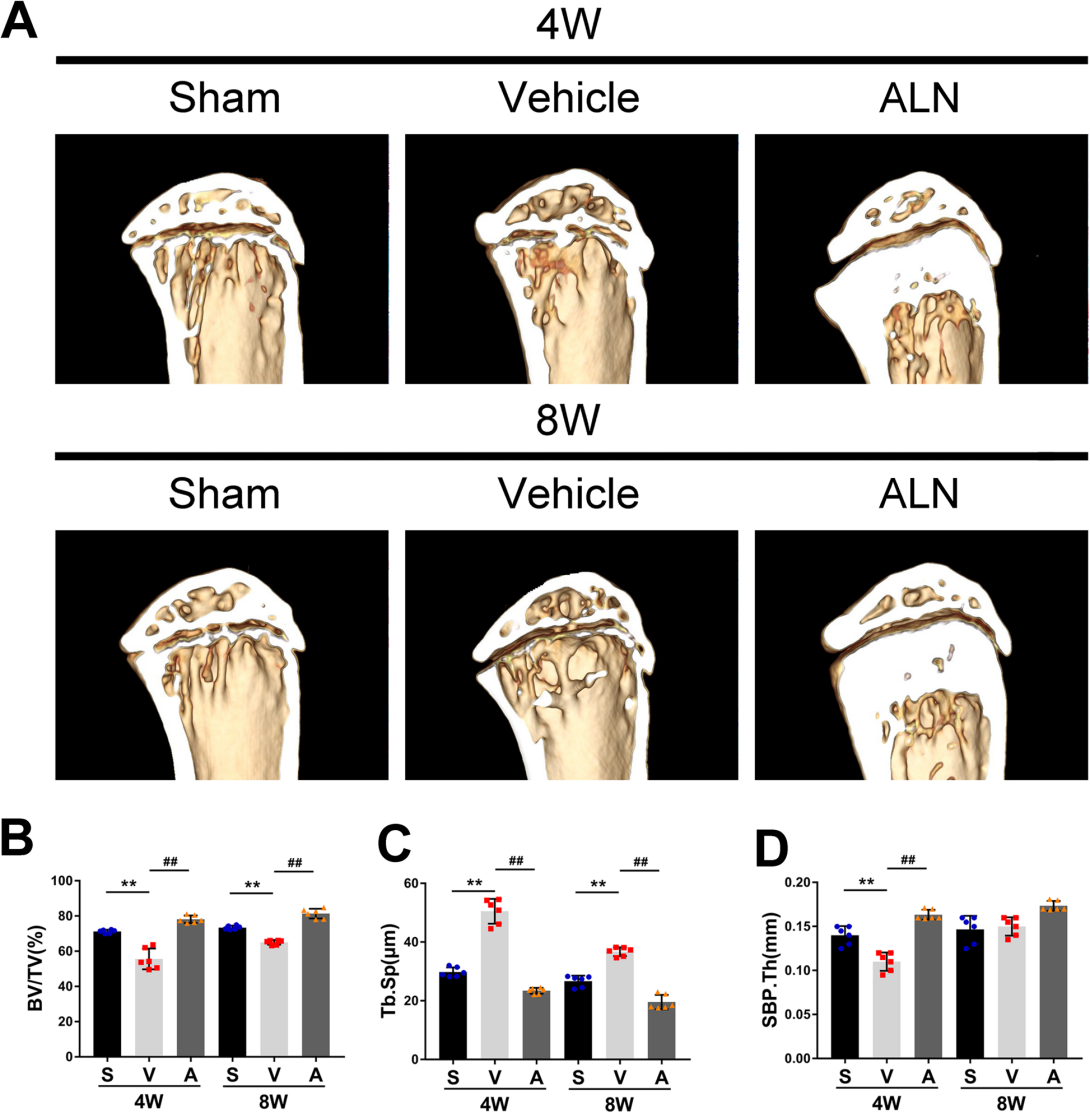
(**A**) micro-CT images of sagittal views of the subchondral bone medial compartment at 4 weeks and 8 weeks after the sham operation or ACLT surgery. (**B** − **D**) Quantitative analysis of micro-CT parameters of the tibial subchondral bone: bone volume fraction (BV/TV) (**B**), subchondral bone plate thickness (SBP.Th) (**C**) and trabecular separation (Tb.Sp)(**D**). Vehicle = ACLT surgery treated with vehicle. ALN = ACLT surgery treated with alendronate. Numerical data are presented as means ± SD; ^**^p < 0.01 compared to the sham group, ^##^*p* < 0.01 compared to the vehicle group

Micro-CT was used to further study the effect of ALN on the microstructure of subchondral bone and its correlation with articular cartilage protection. Inhibition of osteoclasts improved the microstructure of the subchondral bone. BV/TV in the vehicle group was significantly lower than that of the sham group 4 weeks after the surgery, while the value in the ALN group was significantly higher. Similar results were obtained in the data 8 weeks after the surgery (Fig. 3 A, B). The subchondral bone plate closely related to articular cartilage decreased significantly in the vehicle group compared to the sham group at 4 weeks post-operatively, while the ALN group maintained the thickness of the subchondral bone, which indicated that ALN eliminated the influence of bone resorption on the thickness of the subchondral bone plate. Compared to the 4-week sham group, the values of the vehicle group decreased first and then increased at 4, and 8 weeks, which showed that the subchondral bone plate changed from bone absorption to bone formation (Fig. 3 A, C). The increased Tb. Sp indicated that bone catabolism was greater than anabolism. Compared to the sham group at 4 and 8 weeks after surgery, the vehicle group increased significantly, and the ALN group abrogated the effect of modeling. (Fig. 3 A, D). These data indicated that the inhibition of osteoclasts by ALN could attenuate the process of abnormal bone remodeling of the subchondral bone after modeling.

### Effect of LIF secreted by osteoclasts on the abnormal bone remodeling of the subchondral bone

**Fig. 4:**
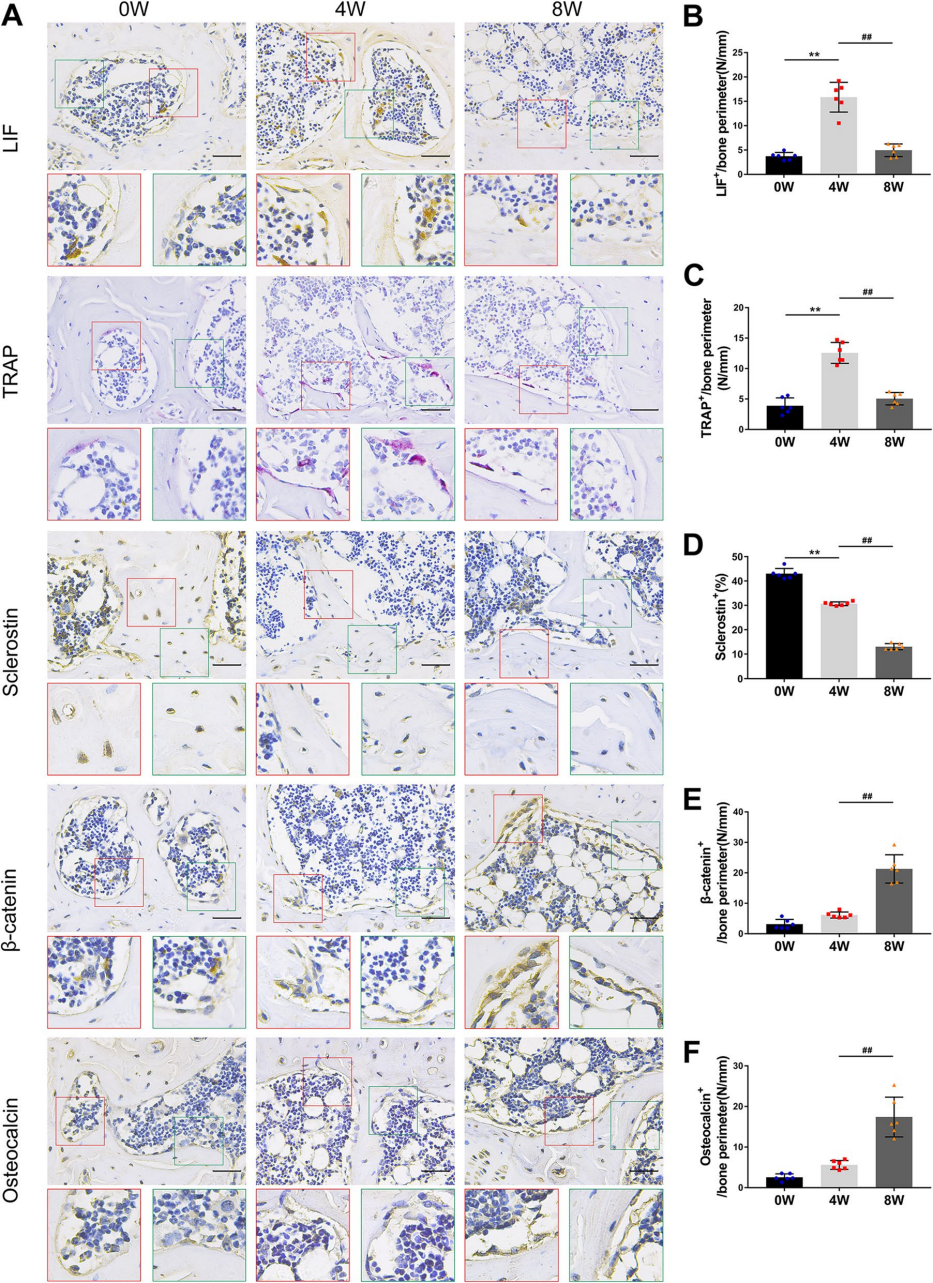
(**A** − **F**) Immunostaining and quantitative analysis for LIF (**A**, **B**), TRAP (**A**, **C**), sclerostin (**A**, **D**), β-catenin (**A**, **E**) and osteocalcin (**A**, **F**) in sagittal sections of the subchondral bone medial compartment at 4 weeks and 8 weeks after ACLT surgery. Scale bar, 50 μm. Means ± SD; ^**^p < 0.01 compared to the vehicle group at 0 weeks, ^##^*p* < 0.01 compared to the vehicle group at 4 weeks

**Fig. 5:**
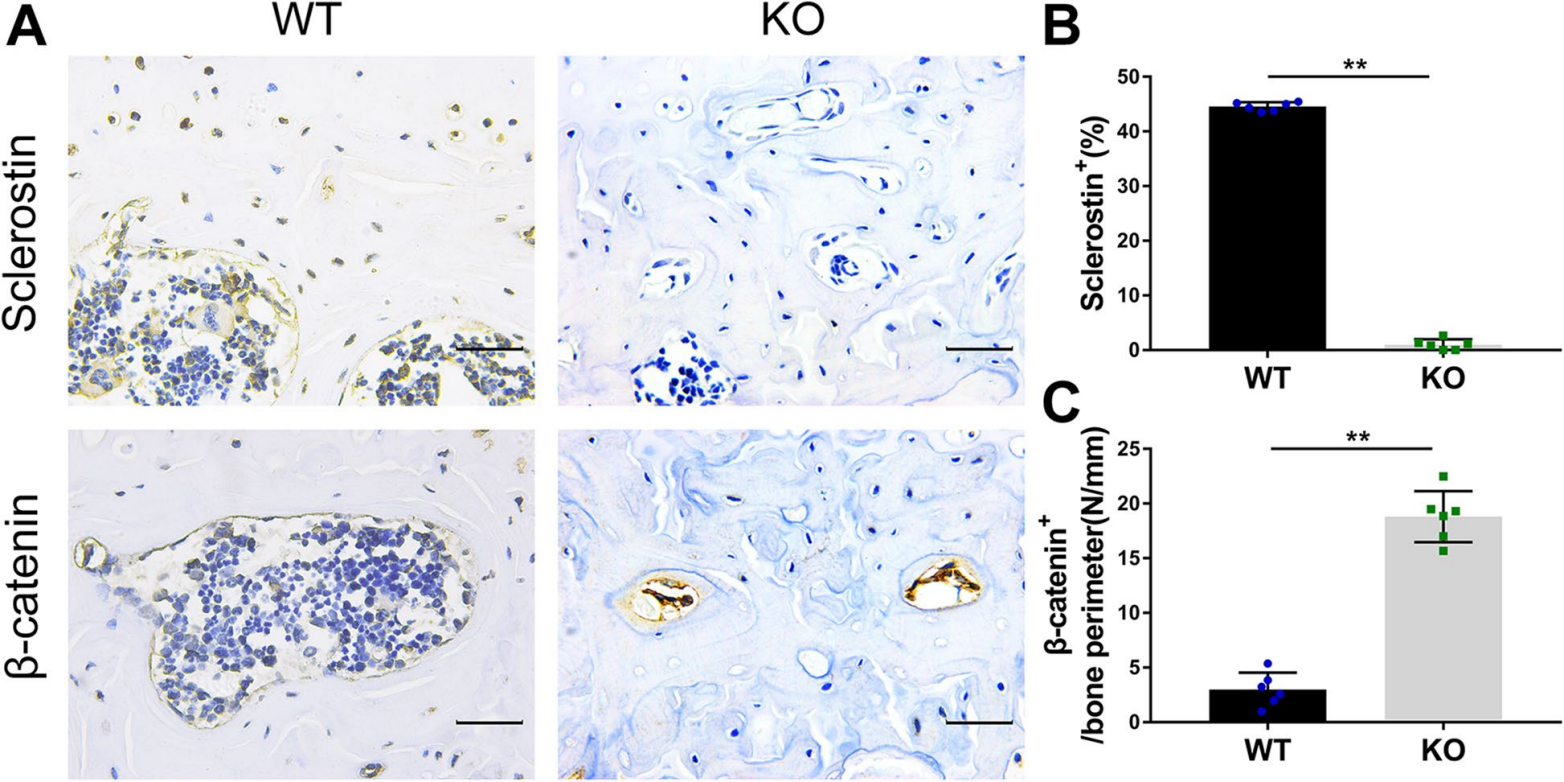
(**A** − **C**) Immunostaining and quantitative analysis for sclerostin (**A**, **B**) and β-catenin (**A**, **C**) in sagittal sections of the subchondral bone medial compartment at 4 weeks after sham surgery. WT = Wild type sham, KO = *SOST* knockout sham mice. Scale bar, 50 μm. Means ± SD; ^**^p < 0.01 compared to the sham group

Immunohistochemical staining was used to study the effect of clastokine LIF on the microstructure of the subchondral bone. Compared to the vehicle group at 0 weeks after the operation, the LIF positive cells of osteoclasts in the vehicle group increased significantly 4 weeks later but decreased after 8 weeks (Fig. 4 A, B). The TRAP staining showed similar results (Fig. 4 A, C). At 0, 4, and 8 weeks after the surgery, the number of sclerostin-positive cells in the vehicle group decreased gradually (Fig. 4 A, D), while the number of β-catenin-positive cells in osteoblasts increased gradually, which was similar to the results of osteocalcin (bone formation index) of osteoblasts (Fig. 3 A, E, F). Compared with the *SOST* KO group, the number of β-catenin-positive cells in the sham group was significantly higher than that in the *SOST* KO group, indicating that sclerostin negatively regulates the expression of β-catenin in the osteoblasts (Fig. 5 A − C). These results suggested that osteoclast activation and LIF expression might downregulate sclerostin, and then activate the β-catenin signal, which results in increased bone formation and slower bone remodeling.

### ALN inhibited osteoclasts and attenuated abnormal bone remodeling of the subchondral bone

**FIG. 6:**
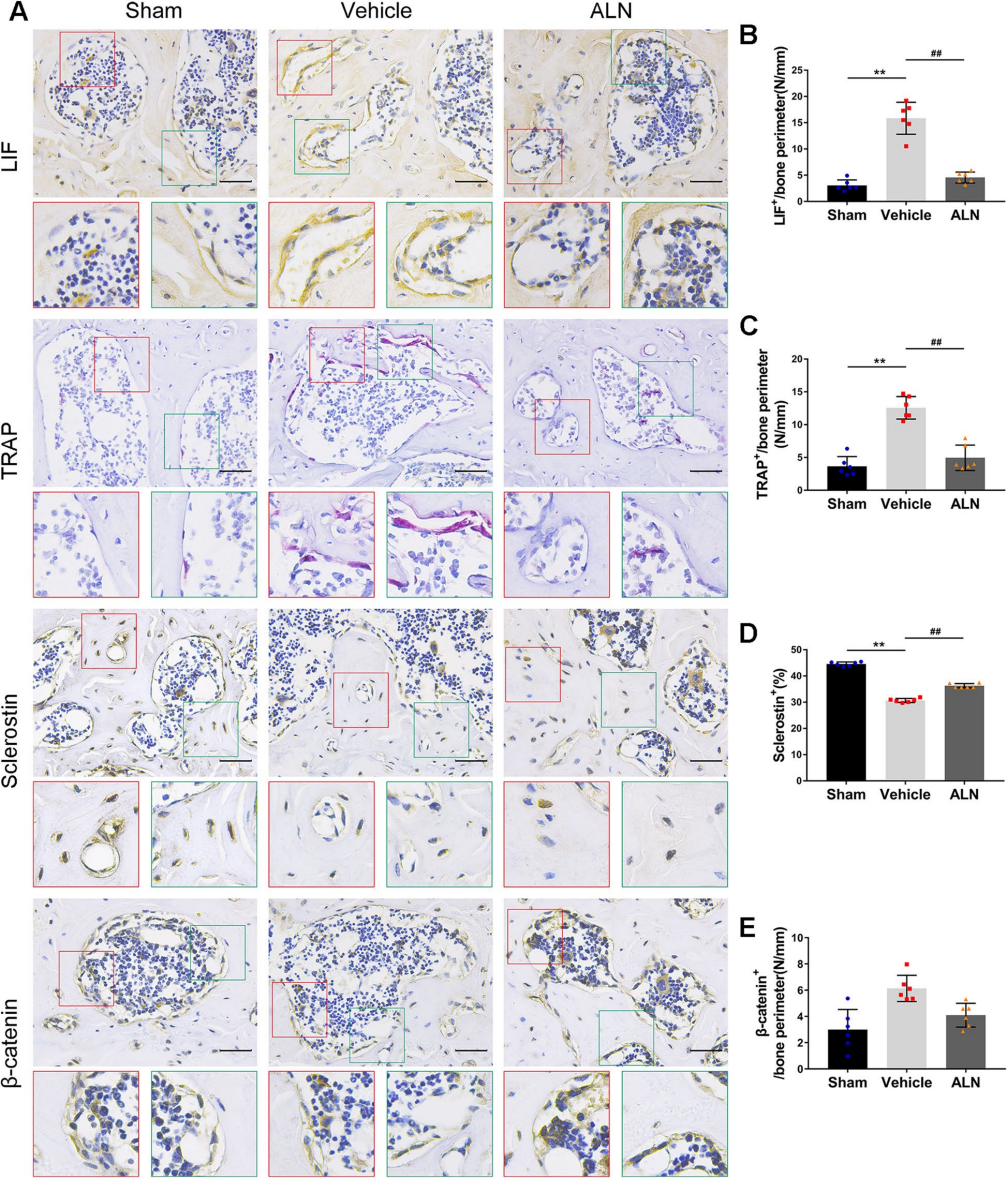
(**A** − **E**) Immunostaining and quantitative analysis for LIF (A, B), TRAP (**A**, **C**), sclerostin (**A**, **D**) and β-catenin (**A**, **E**) in sagittal sections of the subchondral bone medial compartment at 4 weeks after ACLT surgery. Scale bar, 50 μm. Means ± SD; ^**^p < 0.01 compared to the sham group, ^##^*p* < 0.01 compared to the vehicle group

Furthermore, the study investigated the effect of ALN on osteoclasts and found a decrease in LIF secretion on the microstructure of subchondral bone. The results showed that the number of LIF positive cells in the vehicle group was significantly higher than that in the sham group at 4 weeks after the operation, while the ALN group attenuated the difference (Fig. 6 A, B). This was similar to the results of TRAP staining of osteoclasts (Fig. 6 A, C). However, the number of osteoclasts has affected the expression of sclerostin. At 4 weeks after the operation, sclerostin in the vehicle group was significantly decreased, while that in the ALN group was close to that in the sham group (Fig. 6 A, D). The number of β-catenin-positive cells in the vehicle group was higher than that in the sham group. Compared to β-catenin in the sham group, the bone formation ability was increased in the ALN group (Fig. 6 A, E). These results showed that by inhibiting the number of osteoclasts and reducing the expression of LIF, sclerostin could be restored, maintained the microstructure of the subchondral bone, and attenuated the process of abnormal bone remodeling.

## Discussion

The progression of OA disease is known to be characterized by subchondral bone loss in the early stage and osteosclerosis in the late stage. A large amount of evidence suggests that subchondral bone remodeling plays a key role in the occurrence and development of OA [16]. This study demonstrated the potential mechanism of abnormal remodeling of the subchondral bone through the establishment of an unstable OA model and drug intervention. Specifically, We speculate that differentiated osteoclasts secrete a large amount of LIF, which inhibits the sclerostin in the surrounding osteocytes, and then activates the Wnt/β-catenin pathway to change bone turnover, while ALN inhibits osteoclast activity, reducing the secretion of LIF, protecting the articular cartilage, and attenuating the process of OA.

The microstructure of the subchondral bone changes with the development of OA. Research has shown that in the early stages of OA, young and middle-aged (27–56 years old) patients have elevated subchondral bone resorption markers [17]. The increase in the bone resorption area elevates the risk of overlying cartilage damage [18]. MMP-13 can cause degradation of articular cartilage [19]. Col X is a specific marker of chondrocyte hypertrophy [20]. In this study, the ACLT model was used to increase the abnormal mechanical stress of the knee joint. The OARSI histology score, MMP-13, and Col X indicators in the vehicle group increased. At the same time, the micro-CT of the subchondral bone showed that compared to the sham group, the BV/TV, and SBP. Th of the vehicle group decreased, while Tb. Sp increased. We found that when the basic cartilage structure was complete and there was no obvious damage, the microstructure of the subchondral bone changed to a large extent, mainly bone resorption. Compared to the vehicle group, the OARSI histological score, MMP-13, and Col X indicators were significantly improved after ALN treatment, and the micro-CT related parameters of subchondral bone were close to those of the sham group. These results indicate that the bone resorption of subchondral bone is increased in the early stage of OA, and the cartilage is progressively damaged. After inhibiting the activity of osteoclasts, the characteristics of subchondral bone are maintained, and the articular cartilage is protected.

The bone remodeling of subchondral bone mainly depends on the signal conduction between osteoclasts, osteoblasts, and osteocytes. The process involves bone resorption by osteoclasts on the surface of the bone and then bone formation. It is reported that the bone matrix releases transforming growth factor β (TGF-β) and insulin-like growth factor I (IGF-I) during bone resorption, which induces the migration of osteoblast precursors to the site of bone resorption and promotes bone formation [21, 22]. Previous studies have shown that TGF -β1 can induce the expression of CXCL16 and leukemia inhibitory factor, and regulate the migration of osteoblast progenitor cells to recover bone loss during bone turnover and absorption [23]. Recent studies have shown that LIF secreted by mature and activated osteoclasts can promote bone formation by inhibiting the expression of sclerostin and upregulating Wnt/β-catenin. LIF can bind to the cytokine binding region (CHR) of glycoprotein 130(gp130) on osteocytes to form the LIF/gp130/LIFR complex and downregulate its sclerostin [24, 25]. In this study, the TRAP levels increased first and then decreased in the vehicle group 4, and 8 weeks after the operation, which was consistent with the results of the subchondral bone turnover during OA progression. This was similar to the change in the LIF levels. The sclerostin level gradually decreased. After ALN intervention, the osteoclast activity was inhibited, corresponding to the decrease in LIF expression, and sclerostin was recovered. We speculate that the change in LIF may be have a potential effect in the turnover of subchondral bone abnormal remodeling in OA mice. In addition, other osteoclast-derived factors can also interact with LIFR to stimulate bone formation, such as cardiotrophin 1(CT-1) and oncostatin M (OSM). However, the results indicated that LIF, CT-1, and OSM might not be highly involved in inhibiting the expression of sclerostin in the state of high bone turnover [10].

Sclerostin binds to the extracellular domain of low-density lipoprotein receptor-related protein 5(LRP5) and antagonizes the Wnt signaling, which has a negative regulatory effect on the osteoblastic cells [26]. Our team has previously confirmed in human clinical samples that sclerostin negatively regulates β-catenin in the subchondral bone of OA patients [27]. The relationship between them was further proved by the *SOST* KO mice. In this study, the expression of β-catenin and osteocalcin increased gradually with the change of sclerostin in the vehicle group 4, and 8 weeks after the operation, and the abnormal bone remodeling of the subchondral bone transformed into bone formation, which was also supported by the micro-CT parameters. The deficiency of sclerostin can increase the osteogenic activity of subchondral bone and aggravate OA in mice [28]. This indicates that slow bone remodeling can accelerate the progression of OA, while ALN can attenuate the turnover process.

ALN can eliminate the abnormal increase in bone remodeling in early OA. It reduces the number of osteoclasts and promotes their apoptosis by preventing the recruitment, and fusion of pre-osteoclasts, to keep osteoclasts away from the bone surface [29]. Some studies have shown that ALN can increase the degree of mineralization in bone by promoting the combination of hydroxyapatite crystals on the bone remodeling site [30]. Our results show that ALN maintains the structure of the subchondral bone, reduces the effect of knee instability on LIF, and protects the articular cartilage. However, we also observed that long-term application of ALN may lead to excessive mineralization of bone. And clinically, the side effects of high-dose ALN would likely result in osteonecrosis [31]. In addition, ALN has no significant effect on the amount of sclerostin in osteocytes [32]. This also indirectly shows that ALN inhibits the number of osteoclasts and reduces the expression of the osteoclast-derived factor, LIF, thereby affecting the changes in sclerostin.

In conclusion, this study showed that subchondral bone remodeling increased in the early stage of OA, and a large number of osteoclasts secreted LIF to negatively regulate the expression of sclerostin in osteocytes, and then upregulated WNT/β-catenin to promote bone formation and restore coupled bone remodeling. The application of ALN to inhibit osteoclasts weakened the promotion of LIF on the early abnormal bone remodeling of OA and attenuated osteoarthritis. However, this study has certain limitations, and more in vitro and in vivo experiments are needed for further verification to determine the regulatory mechanism of LIF in abnormal bone remodeling. This study provides a deeper understanding of the role of clastokine LIF in abnormal bone remodeling in early OA.

## Supplementary Information

**Additional file 1.** The study was carried out in compliance with the ARRIVE guidelines.

## Data Availability

The datasets used during the current study are available from the corresponding author on reasonable request.

## Abbreviations

OA: Osteoarthritis
ALN: Alendronate
LIF: Leukemia inhibitory factor
SOST: Sclerostin
KO: Knockout
BV/TV: Bone volume/total tissue volume
SBP.Th: Subchondral bone plate thickness
Tb.Sp: Trabecular separation
HRQOL: Health-related quality of life
GWAS: Genome-wide association studies
M-CSF: Macrophage colony-stimulating factor
RANKL: Receptor activator for nuclear factor-κB ligand
ACLT: Anterior cruciate ligament transection
TRAP: Tartrate-resistant acid phosphatase
MMP-13: Matrix metalloproteinase 13
Col X: Type X collagen
TGF-β: Transforming growth factor β
IGF-I: Insulin-like growth factor I
CHR: Cytokine binding region
gp130: Glycoprotein 130
CT-1: Cardiotrophin 1
OSM: Oncostatin M
LRP5: Lipoprotein receptor-related protein 5
