# Supplementary material for: Osteoclasts secrete leukemia inhibitory factor to promote abnormal bone remodeling of subchondral bone in osteoarthritis
Source: BMC Musculoskelet Disord. 2022 Jan 25;23:87. doi: 10.1186/s12891-021-04886-2 (PMC8790929; doi:10.1186/s12891-021-04886-2)
Supplement: Supplementary file 1 — Additional file 1. The study was carried out in compliance with the ARRIVE guidelines. [file 12891_2021_4886_MOESM1_ESM.docx]

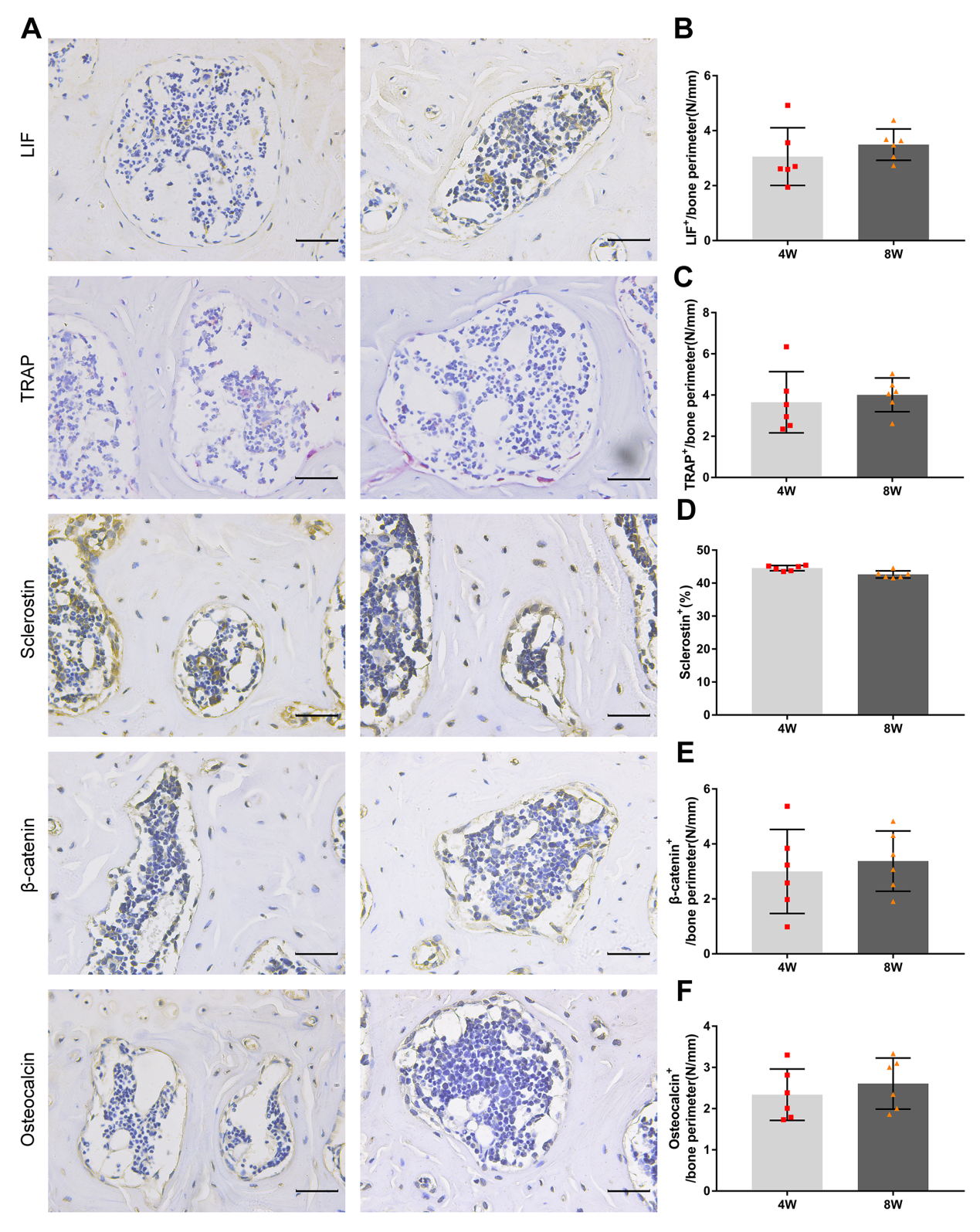


Supplementary Figure(A−F) Immunostaining and quantitative analysis for LIF (A, B), TRAP (A, C), sclerostin (A, D), β-catenin (A, E) and osteocalcin (A, F) in sagittal sections of the subchondral bone medial compartment at 4 weeks and 8 weeks after sham surgery. Scale bar, 50 μm. Means ± SD; ^**^p <0.01 compared to the sham group at 4 weeks.
